# Study of the Structural, Morphological, Strength and Shielding Properties of CuBi_2_O_4_ Films Obtained by Electrochemical Synthesis

**DOI:** 10.3390/ma16227241

**Published:** 2023-11-20

**Authors:** Dauren B. Kadyrzhanov, Malik E. Kaliyekperov, Medet T. Idinov, Artem L. Kozlovskiy

**Affiliations:** 1 Engineering Profile Laboratory, L.N. Gumilyov Eurasian National University, Astana 010008, Kazakhstan; dauren.kadyrzhanov@mail.ru (D.B.K.);; 2 NJSC, Shakarim University of Semey, Semipalatinsk 071400, Kazakhstan; 3 Laboratory of Solid State Physics, The Institute of Nuclear Physics, Almaty 050032, Kazakhstan

**Keywords:** thin films, electrochemical deposition, shielding materials, protective films, γ-radiation

## Abstract

In this research, the formation processes of CuBi_2_O_4_ films were examined using atomic force microscopy, energy dispersive analysis and X-ray diffraction analysis methods. The films were synthesized through electrochemical deposition from sulfuric acid solutions at a potential difference of 3.5 V. The duration of film growth was set to between 10 and 90 min to assess the possibility of controlled film growth and preserve the stability of their structural properties during growth over an extended period. An analysis of the data obtained by X-ray diffraction revealed that the resulting film samples are highly ordered structures with a tetragonal CuBi_2_O_4_ phase. The results of the connection between the thickness of CuBi_2_O_4_ films and strength properties depending on the time of film deposition were obtained. The results of the shielding efficiency of low-energy γ-quanta using CuBi_2_O_4_ films were obtained.

## 1. Introduction

Over the past few years, there has been an increasing focus on the issue of protection against the adverse effects of ionizing radiation, in particular, X-ray and gamma-ray radiation, which have a high penetrating capacity, in contrast to heavy ions [1,2,3]. At the same time, the nature of the interaction between X-ray and gamma radiation and materials, including living organisms, based on ionization processes, can lead to detrimental consequences. So, for living organisms, prolonged exposure to ionizing radiation leads to the occurrence of mutations in DNA, leading to the formation of cancer cells or tumors that can lead to death [4,5]. In the case of microelectronic devices, exposure to ionizing radiation can lead to equipment failure or malfunctions due to electrical breakdowns or failure of transmitting or connecting electronic assemblies due to radiation-induced damage. At the same time, the problem of protection against the effects of ionizing radiation is becoming more acute annually due to the expansion of the range of application of various types of ionizing radiation in everyday life as well as the increase in radiation-generating installations that are used for medical purposes, the energy sector, etc. [6,7,8,9].

In this regard, large resources of leading scientific organizations around the world are directed towards searching for new ways to protect against the negative effects of ionizing radiation. The principles of creating protective materials to reduce the negative effects of ionizing radiation are based on the key principles of the As Low As Reasonably Achievable (ALARA) concept adopted by the International Commission on Radiation Protection, the main goal of which is to minimize the negative effects of radiation and various types of ionizing radiation [10,11,12]. This concept is based on the principle of reduction in the impact of ionizing radiation by any possible means, as well as the possibility of optimizing protection using various types of protective materials [13,14]. Much attention in the search for new types of protective materials is paid to various composite films containing various elements, including light elements, heavy or rare earth elements as well as their mixed compositions [15,16]. Interest in these materials is due to the possibility of combining the composition of the obtained films, which makes it possible to increase the shielding efficiency due to differences in the absorbing ability of various elements, as well as the possibility of varying the thickness of the films by changing the conditions for their production [17,18]. Also, the use of various polymeric materials as substrates, in particular polyimide and polycarbonate films, which have good indicators of flexibility and resistance to heat, makes it possible to create protective composite materials with good flexibility, which opens up opportunities for coating complex profile surfaces with protective materials characteristic of a large number of microelectronic devices [19,20,21,22].

Based on this, the key aim of this work is to study the applicability of the electrochemical synthesis method with a variation in the deposition time to obtain thin films based on copper and bismuth compounds as well as to evaluate their applicability as protective materials for γ-radiation shielding [23,24]. Films based on CuBi_2_O_4_ have great prospects for use as protective materials for shielding the negative effects of ionizing radiation, in particular, X-ray and gamma radiation, which have a high penetrating ability [25,26]. At the same time, these films deposited on polymer substrates are highly flexible and resistant to peeling, which, in turn, will allow them to be used, if necessary, as protective materials in conditions of complex geometry of objects that require protection from the adverse effects of ionizing radiation. The choice of the electrochemical deposition method makes it possible to obtain such films not only under laboratory conditions but also to scale up this technology for the industrial production of such films. Interest in the use of new types of materials as protective shields to reduce the intensity of ionizing radiation is due to the need to increase the level of safety when working with ionizing radiation sources. Recently, significant attention has been directed towards advancements in producing various composite shielding materials. These include amorphous glasses or ceramics [27,28,29,30]; polymer matrices embedded with rare-earth metal particles or oxide inclusions (such as iron oxide and bismuth) [31,32]; and composite multilayer films, which are primarily used for electromagnetic radiation shielding [33,34]. At the same time, the assessment of the shielding efficiency of these films is determined both during full-scale experiments using various reference sources of ionizing radiation (Co^57^, Co^60^, Na^22^ and Cs^137^) [35,36] and by simulation of the processes of ionizing radiation interaction with matter using program code based on the Monte Carlo method [37,38]. In this regard, the proposed compositions are in the form of polymer films coated with metal oxide films based on CuBi_2_O_4_, which, due to the elasticity and flexibility of the polymer base, as well as the prospects for shielding metal oxide films, can be considered as one of the candidate shielding materials and can be used to protect against the adverse effects of ionizing radiation in the case of shielding objects with complex geometric shapes that require the use of sufficiently flexible materials.

## 2. Materials and Methods

### 2.1. Synthesis of Thin Film Samples

As a method for obtaining thin CuBi_2_O_4_-based coatings, the electrochemical deposition method was developed by altering the deposition time, which made it possible to obtain films of various thicknesses. The synthesis was carried out at an applied potential difference of 3.5 V. Coatings were synthesized using polymer films based on polyethylene terephthalate, which has high flexibility and inertness to most acids, as well as dielectric properties. Before electrochemical deposition, a thin layer of gold was deposited on the surface of the polymer film due to its dielectric nature by magnetron sputtering, with a thickness not exceeding 30 nm. Electrochemical cells made of polylactide (PLA), a thermoplastic used in the electrochemical industry, were used for the deposition. The following chemicals were used to prepare the electrolyte solution: CuSO_4_·5H_2_O (238 g/L), Bi_2_(SO_4_)_3_ (10 g/L) and H_2_SO_4_ (21 g/L). Deposition was monitored using chronoamperometry. The deposition time was chosen in the range from 10 to 90 min. To control the uniformity of deposition over time, a chlorine–silver reference potential was used.

### 2.2. Characterization of Properties Depending on Synthesis Conditions

Analysis of the structural features of the synthesized films depending on the deposition time was carried out using the method of X-ray diffraction analysis on a D8 Advance ECO powder X-ray diffractometer (Bruker, Mannheim, Germany). The X-ray diffraction patterns were taken in the Bragg–Brentano geometry in the angular range of 2θ = 35 – 75° with a step of 0.03°, and the recording time of the spectrum at a point was 1 sec. Copper radiation with a wavelength of λ = 1.54 Å was used as X-ray radiation. To interpret the obtained diffraction patterns, as well as to analyze the structural parameters, the DiffracEVA v.4.2 software was used, which makes it possible to determine the degree of structural ordering as well as the change in the crystal lattice parameters.

The morphological features of the synthesized films were studied using the method of atomic force microscopy (AFM) (AIST-NT, Chernogolovka, Russia) by constructing 3D images of the surface in order to determine the structural features and grain formation mechanisms.

### 2.3. Study of Strength Characteristics

The indentation method was used to determine the strength characteristics of the synthesized CuBi_2_O_4_ thin films depending on the preparation time. A Vickers diamond pyramid was used as an indenter; measurements were carried out on a Duroline-M microhardness tester (Metkon, Osmangazi/Bursa, Türkiye). The tests were carried out at a constant load on the indenter with a force of 0.1 N. The pressing time on the sample was 15 s, and after measurements the indentations on the surface of the films were studied in order to establish the geometric dimensions of the diagonals of the indenter traces and calculate the hardness values. To determine the Vickers hardness, formula (1) was used [39]:

(1) $$HV=1.854\frac{P}{d^{2}}$$

*P* is the load on the indenter, *N*; *d* is the diagonal size of the indenter print. The average value of the hardness value was determined with serial tests, consisting of 10–15 successive indentations and determinations of hardness values.

### 2.4. Determination of the γ-Radiation Shielding Efficiency Using These Films

**Figure 1:**
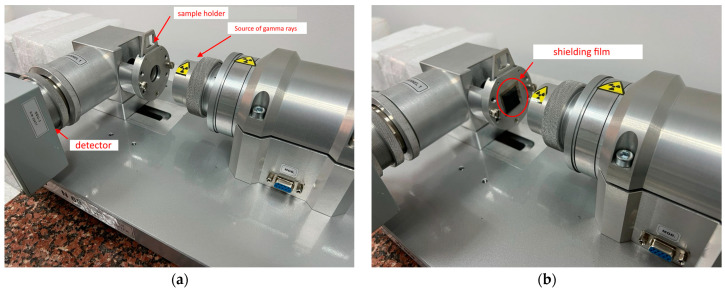
Image of the equipment used for conducting shielding experiments, indicating the arrangement of films used as protective shields: (**a**) scheme of the main nodes for detection and determination of shielding characteristics; (**b**) placement of the shielding film in the spectrometer.

The shielding scheme consists of a source of γ-quanta fixed in a special lead container, having one hole with a diameter of 10 mm through which γ-quanta are emitted and recorded during passage through the target using a detection system based on a NaI scintillation detector. A standard sample of iron (Fe) was used as a target in order to obtain a high-resolution Mössbauer spectrum, which is a Zeeman sextet with uniformly broadened spectral lines. Before all measurements, the device was adjusted and calibrated in order to avoid software errors or inaccuracies in measurements. The spectrum acquisition time was 2 h. Figure 1 reveals images of the Mössbauer spectrometer used to determine the shielding efficiency of synthesized thin films of γ-radiation with a certain fixed energy of 130 keV emitted by a Co^57^ γ-quantum source with an activity of 50 mCi. Shielding experiments were carried out by placing the synthesized thin films in front of a standard sample (as shown in Figure 1b), after which the spectra were taken according to the standard technique.

**Figure 2:**
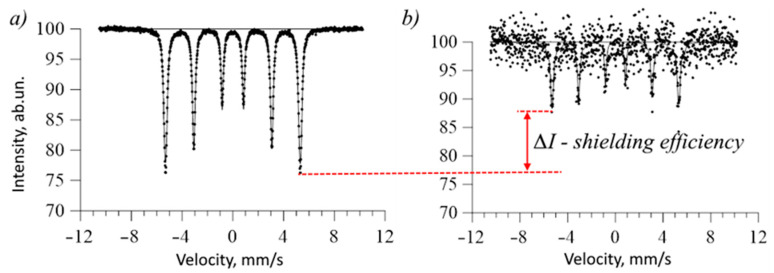
Examples of Mössbauer shielding spectra (these spectra were obtained in laboratory conditions when recording Mössbauer spectra of α-Fe on a spectrometer without a filter from synthesized films and with a film used as a screening film): (**a**) Mössbauer spectrum of a standard sample without shielding; (**b**) Mössbauer spectrum of a standard sample with a thin film synthesized within 60 min.

Shielding efficiency was determined by evaluating the ratio of spectral line intensities, alongside the number of recorded interaction pulses before and after shielding. As an example, Figure 2 illustrates examples of Mössbauer spectra before and after shielding using thin films. The presented Mössbauer spectra were obtained experimentally using the standard α-Fe reference as a sample for recording the spectrum, which is characterized by a classical sextet with well-identified spectral lines of equal intensity (see Figure 2a). Figure 2b demonstrates the Mössbauer spectrum of the α-Fe reference obtained using a protective CuBi_2_O_4_ film placed in front of the sample. Spectral processing of data to construct a graphical representation of the spectra was performed in the program code of Golden Software Grapher v.11.

As can be seen from the given examples of the Mössbauer spectra of standard Fe samples before and after shielding, significant differences are noticeable not only in the intensity of the spectral lines but also in the quality of the spectra themselves. In the case of the Mössbauer spectra of Fe obtained without shielding, the Zeeman sextet has thin spectral lines, with good resolution and description, which indicates good spectrum collection statistics. In the case of using shielding thin films, the quality of the spectrum deteriorates sharply, due to a decrease in statistics, as well as a decrease in the intensity of the lines. An increase in the statistical spread of points on the spectrum indicates a small number of recorded effects associated with the passage and subsequent interaction of γ-quanta with iron nuclei in the standard sample. 

Formula (2) was used to determine the shielding efficiency (RFE) [40,41].

(2) $$RFE=\frac{I_{0}-I_{i}}{I_{0}}\times 100\%$$

where *I*_0_ and *I_i_* are the intensity values before and after shielding.

Formula (3) was used to estimate the linear attenuation coefficient [40,41].

(3) μ=lnI0Id

where *d* is the film thickness. 

The half-attenuation thickness value (∆_1/2_) was calculated using Formula (4) [40,41].

(4) $${∆}_{1/2}=\frac{ln2}{µ}$$

The mass attenuation coefficient depending on the thickness of the synthesized films was calculated using Formula (5) [40,41].

(5) $${µ}_{m}=\frac{µ}{\rho }$$

where *ρ* is the density of samples of thin CuBi_2_O_4_ films.

The mean free path (*MFP*) value was calculated using the Formula (6) [40,41].

(6) $$MFP=\frac{1}{\mu }$$

## 3. Results and Discussion 

### 3.1. Characterization of the Obtained Films Depending on the Deposition Time

**Figure 3:**
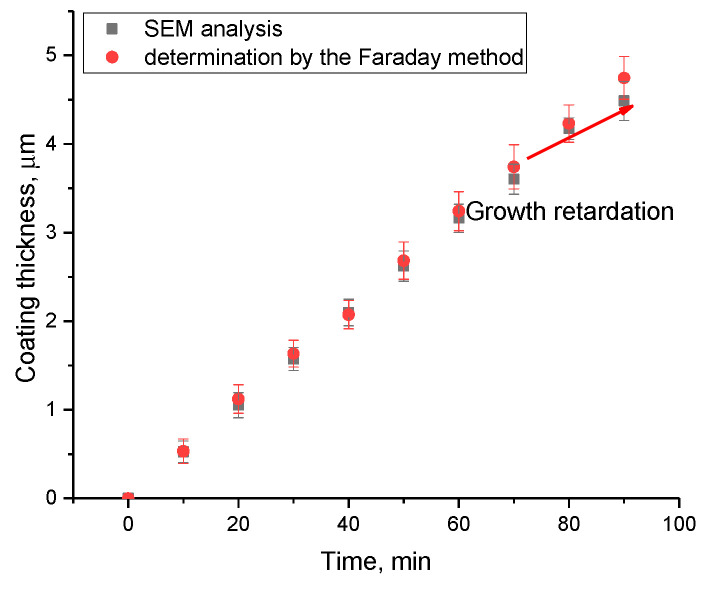
Dependence of the change in the thickness of the synthesized thin films on the deposition time.

The thickness of the synthesized films depending on the deposition time was determined via methods for determination of the thickness by evaluation of the side cleavages using scanning electron microscopy, as well as estimated thickness calculations based on determination of the current density and deposition time using the Faraday method. The results of the comparative analysis are presented in Figure 3. 

**Figure 4:**
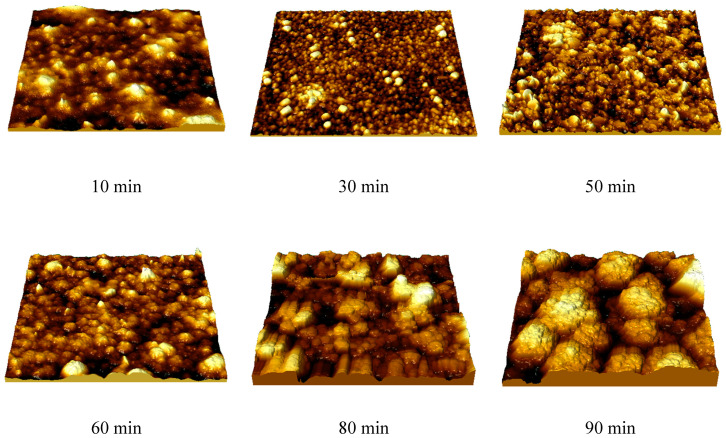
The results of 3D reconstruction of morphological features of synthesized thin films.

As is evident from the data presented, the alteration in film thickness depending on the deposition time, determined using two methods, has good agreement, alongside a linear dependence of the growth in thickness with time, up to 70 min of deposition. In the case of film deposition at times above 70 min, a reduction in film thickness is observed, which indicates a slowdown in film growth. Such a slowdown can be explained by the effects of depletion of sulfuric acid solutions with a long deposition time, which is also evidenced by the current density decrease after 70 min by 5–7% compared to the initial values of current density. The reduction in the film thickness growth rate after 70 min (“growth retardation”), indicated in Figure 3, is attributed to the depletion of electrolyte solution and has a similar behavior for electrochemical processes of metal reduction or their deposition on the surface of various materials during long-term deposition [42]. The observed “growth retardation” stems from the ion concentration reduction in the electrolyte solution, resulting in a slowdown in the formation of the crystalline structure of the films and, as a consequence, a decline in the growth rate of the thickness of the synthesized films. At the same time, a rise in the deposition time above 50 min, according to the data of atomic force microscopy, results in a change in the morphology of the emerging coatings with the formation of large agglomerates, which are clusters of grains (see data in Figure 4), which results in the appearance of film heterogeneity and the roughness growth. 

As can be seen from the data presented, at short deposition times (up to 10 min), the main growth of films consists of the formation of nucleation centers on the surface of the substrate, which act as “anchor centers” near which film growth occurs. As a result of this growth mechanism, according to the presented 3D images, there is a pronounced difference in both the geometry of the grains (the formation of large and small grains) and differences in the height profile of the resulting films. As the deposition time extends (30–60 min), stabilization of film growth, expressed in the formation of larger grains (due to the coarsening of smaller grains associated with crystallization during the growth process), alongside the formation of films that are more uniform in thickness (the roughness and waviness of the films are reduced by minimizing the variation in the sizes and shapes of crystallizing grains), is observed. At deposition times of 80–90 min, the formation of large agglomerates of grains, alongside an elevation in the waviness and roughness of the resulting films with growing deposition time, is observed. This pattern may be attributed to the depletion of the electrolyte solution and minor reductions in current density, both of which suggest a slowdown in the film growth rate. 

**Figure 5:**
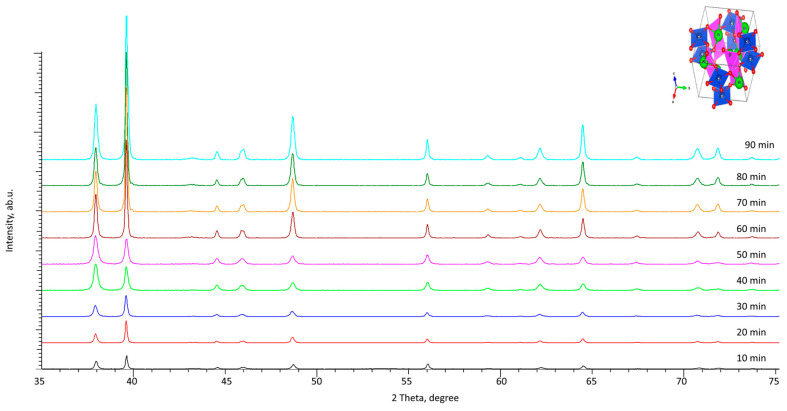
The results of X-ray diffraction of Cu–Bi thin films depending on the deposition time (inset shows the crystal lattice type of CuBi_2_O_4_).

Figure 5 demonstrates the dynamics of changes in X-ray diffraction patterns of synthesized Cu–Bi-based films by electrochemical deposition depending on the deposition time. An analysis of the obtained diffraction patterns revealed that the experimentally obtained diffraction reflections correspond to the tetragonal CuBi_2_O_4_ phase (PDF-00-042-0334), and a growth in the deposition time does not lead to the appearance of additional reflections, which indicates the absence of phase transformations or other structural changes associated with the formation of impurity inclusions or polymorphic transformations. Table 1 presents the results of alterations in the crystal lattice parameters and volume depending on the deposition time. 

The provided data on the variation in crystal lattice parameters demonstrate that a longer deposition time results in the structural ordering degree growth, which is expressed in a decrease in parameters (the most pronounced alterations are observed when the deposition time is changed from 10 to 60 min), which results in a reduced lattice volume and, consequently, compaction of the crystal lattice. The crystal lattice volume reduction results in a rise in ceramic density and, as a consequence, a decline in structural distortions and deformations in the crystal lattice. In the case of deposition times above 60 min, there is a decrease in the reduction rate in crystal structure parameters, which indicates stabilization of the crystal structure formation processes with a further film thickness growth. Moreover, the tetragonality degree (ratio of parameters c/a) with variation in deposition time changes slightly from 0.678 (for samples obtained with 10 min of deposition) to 0.676 (for deposition times above 50 min). Such tetragonality value alterations indicate a marginal effect of deposition time on the change in the compactness of the arrangement of atoms at the crystal lattice sites. Such small changes are attributable to small structural modifications in the crystal lattice parameters during extended deposition times.

**Figure 6:**
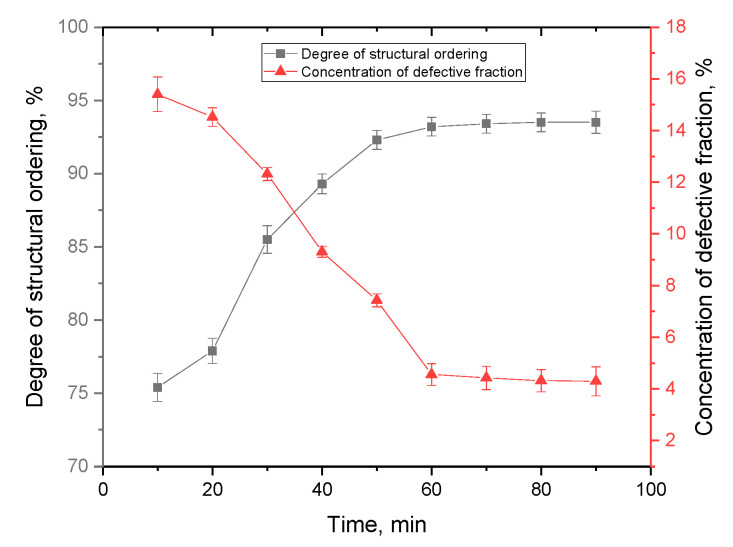
The results of changes in the structural ordering degree and the concentration of defective fraction in films depending on the deposition time.

The alteration results of the structural ordering degree are shown in Figure 6 in comparison with the concentration of the defective fraction in the crystal lattice volume, calculated on the basis of changes in the parameters of the crystal lattice and its volume. The concentration of the defective fraction (structural distortions, amorphous inclusions leading to deformation of the crystal lattice, an increase in its volume, etc.) in film samples was calculated using the values of changes in the structural parameters of the crystal lattice (crystal lattice volume) by comparing them with the values characteristic of card values from the PDF-00-042-0334 database. The structural ordering degree (crystallinity degree), in turn, was determined by approximating the areas of diffraction reflections with a set of pseudo-Voigt functions, followed by comparing the contributions of diffraction reflections with the area characteristic of the amorphous component of the films. 

The variation in the structural ordering degree for the films under study contingent on deposition time can be attributed to the fact that when films are formed over a long period of time, the formation of a more ordered crystal lattice is observed, as evidenced by the data presented in Table 1. Moreover, the growth of films over a long period of time is associated with the formation of denser packing of grains, which is due to the filling of the voids between grains (which are activation centers of nucleation) with smaller grains, which is clearly visible in Figure 4. 

The main changes associated with the deposition time are the structural ordering, which is due to the processes of film growth, as well as the strengthening of the films and the change in the size of the formed grains. As can be seen from the data presented, at the growth stage, which is characteristic for the deposition time range of 10–50 min, the structural ordering degree grows, which is associated primarily with the formation of films, as well as their compaction. At the same time, an analysis of the position of the main diffraction reflections indicates that with an increase in the deposition time above 60 min, the degree of structural ordering changes significantly less than with shorter deposition times, which indicates a possible saturation effect, as well as reaching the limit of the degree of structural ordering. Similar dependences of changes on the deposition time are also observed for the concentration of the defective fraction in films, the presence of which is due to deformation distortions of the structure. With a rise in the structural ordering degree with a change in the growth time, the defective fraction is displaced due to the formation of a structurally ordered crystal lattice.

**Figure 7:**
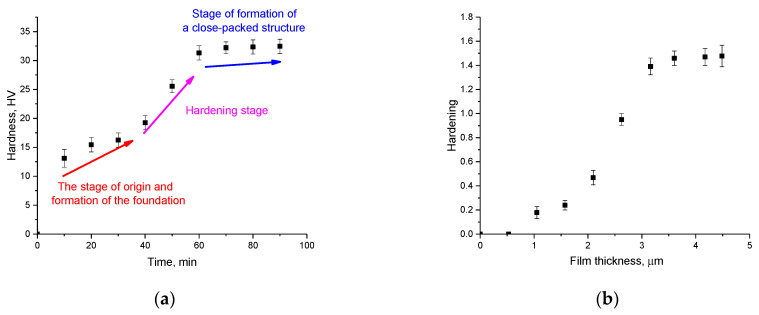
(**a**) The results of changes in hardness depending on the deposition time of thin films; (**b**) the connection between thin film thickness and hardening.

The indentation method was used to determine the strength characteristics of the synthesized CuBi_2_O_4_ thin films depending on the preparation time. The evaluation results of hardness depending on the deposition time are shown in Figure 7. Figure 7a demonstrates the dependence of the change in hardness of the resulting films with varying deposition conditions (time), and Figure 7b reveals the dependence of the hardening (i.e., rise in hardness) of the films with growth in thickness. Thus, this figure reflects the results of alterations in the strength properties of films, alongside the ”saturation“ effect, i.e., at what thickness (or deposition time) the change in film hardness remains practically unchanged.

According to the presented calculations of the hardness value depending on the deposition time of thin films, three main stages of film formation were established, characterized by different effects of hardening and alterations in strength characteristics. The first stage is typical for deposition times in the range of 0–40 min, for which hardness values fluctuate in the range of 13–15 HV, with a slight increase depending on the deposition time. This stage is typical for the nucleation of activation centers, around which the main structure is formed as a result of crystallization processes, as well as the subsequent formation of a massive film on the substrate surface. In this case, low hardness values are due to a poorly bound crystal structure, as well as a small film thickness, under external influences through which it is deformed. 

The second stage is characteristic of a sharp increase in hardness values in the deposition time range from 50 to 70 min due to the effects of hardening and the formation of structurally ordered films. 

The third stage from 70 to 90 min of deposition is characterized by small changes in hardness values of 32–32.5 HV, which indicates the formation of a close-packed structure of films with high strength, which does not change depending on the thickness, which varies with increasing deposition time. 

Figure 7b presents the results of a comparative analysis of changes in the strengthening value and thickness of thin films synthesized depending on the deposition time. This comparative analysis makes it possible to establish the relationship between the indicators of strength characteristics, including hardening and the increase in crack resistance and the thickness of the obtained films. The calculation of the hardening value was made on the basis of changes in the hardness value depending on the deposition time. As the initial value against which the comparison was made, the value of the hardness of the films obtained at a deposition time of 10 min was chosen. The general form of the presented dependence indicates that the main hardening process occurs in the range of deposition times from 0 to 60 min, which are typical for the formation of film thicknesses below 3.5 µm. In the case when the film thickness becomes higher than 3.5 µm, the strengthening value remains constant, which means that the greatest strengthening effect which can be achieved with an increase in the deposition time is no more than 1.6 times that of the initial hardness value. Moreover, in this case, the change in the hardness value at different deposition times indicates the presence of anisotropy in the hardness value, with characteristic low values of the hardness indices at film thicknesses less than 2.0–3.0 µm. 

### 3.2. Conducting Experiments to Evaluate the Use of Synthesized Thin Films of Various Thicknesses for Shielding γ-Quanta

**Figure 8:**
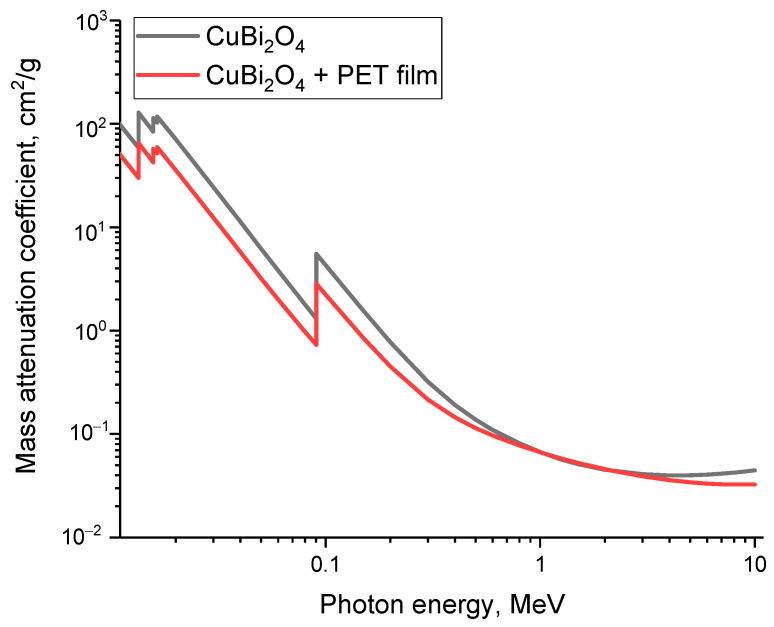
Calculation results of the mass attenuation coefficient obtained using the XCOM program code.

Figure 8 reveals findings of the assessment results of the shielding efficiency of γ-quanta in the energy range from 0.01 to 10 MeV, which covers the main processes of interaction of ionizing radiation with matter (photo effect, Compton effect and the formation of electron–positron pairs). Mass attenuation coefficient (MAC) calculations were obtained using the publicly available XCOM code (https://physics.nist.gov/, accessed on 20 October 2023). For the calculations, two types of structures were used, CuBi_2_O_4_ films and CuBi_2_O_4_ + PET films, which were applied as a substrate to produce films. Analysis of the data obtained indicates a fairly high shielding efficiency (attenuation of the ionizing radiation flux) at low energies of γ-quanta (from 0.01 to 0.5 MeV). Moreover, the decrease is quite sharp with an elevation in the energy of γ-quanta above 1 MeV, which are characterized by processes of formation of electron–positron pairs. The determined relationships between the mass attenuation coefficient (MAC) and the energy of γ-quanta mirror the theoretical calculations for similar quantities in amorphous glasses and ceramics. In these materials, bismuth oxide is frequently used not only for its superior shielding efficiency but also as a reinforcing agent, enhancing the resistance of protective materials to external influences [43,44,45].

Table 2 presents the results of a comparative analysis of the shielding characteristics data obtained during the determination of the shielding efficiency of CuBi_2_O_4_ films obtained after 90 min of deposition with the calculated data obtained using the XCOM program code. The shielding characteristics were determined during experiments on shielding γ-quanta with an energy of 130 keV (Co^57^ source). Calculations were performed using formulas (2)–(6).

As is evident from the data presented, the shielding characteristics calculated using the XCOM program code slightly exceed the experimental values by about 15–20%, which can be explained by factors related to the structural ordering degree and the density of the resulting CuBi_2_O_4_ films, since in the case of theoretical calculations, the density of the selected compound is taken as a standard and is also clearly fixed by the ratio of the elements of the selected compound. Moreover, according to experimental data, the stoichiometric ratio of elements and the density of the resulting films may differ from the theoretically predicted values, which agrees quite well with the data of X-ray diffraction analysis. At the same time, the obtained values of the shielding characteristics of CuBi_2_O_4_ films show good agreement with the results of the shielding parameters of polymer composites [33,34,40], which are considered among the potential materials for shielding low-energy gamma radiation.

## 4. Conclusions

The paper presents the results of the study of the formation of thin CuBi_2_O_4_-based films using the electrochemical deposition method, as well as study of the connection between the coating thickness and strength and shielding characteristics. During the studies, it was found that the formation of films, depending on the deposition time, occurs through the formation of activation growth centers at the initial stage, which serve as the basis for the formation of larger grains, followed by filling the voids between them and the formation of films consisting of small grains with fairly dense packing. During determination of the growth dynamics of the thickness of thin films depending on the deposition time, it was found that the main growth of films at an applied potential difference of 3.5 V occurs at an average rate of 0.05 µm/min, which makes it possible to obtain films or coatings of various thicknesses in a controlled manner. The results of the connection between the thickness of CuBi_2_O_4_ films and strength properties depending on the time of film deposition were obtained. It has been established that the hardening processes associated with structural ordering are most pronounced for samples obtained at deposition times of more than 60 min, which are characterized by the achievement of thicknesses of the order of 3.0–3.5 µm. At the same time, in the case of samples with a thickness of more than 4.0 µm, the hardness value remains constant without significant changes, which indicates the limit of achieving strength in the synthesized films. Preliminary experimental results have shown the possibility of using CuBi_2_O_4_ films as shielding films to reduce the ionizing radiation intensity. Further research will be aimed at determining the resistance of these films to external factors, including aggressive environments.

## Figures and Tables

**Table 1:** Structural parameter data.

Deposition Time, min	Crystal Lattice Parameters, Å	Tetragonality Degree (c/a)	Crystal Lattice Volume, Å^3^
10	a = 8.4242 ± 0.0014, c = 5.7132 ± 0.0015	0.678	V = 405.45
20	a = 8.4224 ± 0.0013, c = 5.7031 ± 0.0016	0.677	V = 404.56
30	a = 8.4193 ± 0.0012, c = 5.7001 ± 0.0017	0.677	V = 404.05
40	a = 8.4167 ± 0.0019, c = 5.6963 ± 0.0015	0.677	V = 403.53
50	a = 8.4153 ± 0.0014, c = 5.6945 ± 0.0012	0.676	V = 403.27
60	a = 8.4146 ± 0.0013, c = 5.6934 ± 0.0018	0.676	V = 403.13
70	a = 8.4142 ± 0.0018, c = 5.6933 ± 0.0015	0.676	V = 403.08
80	a = 8.4141 ± 0.0021, c = 5.6931 ± 0.0013	0.676	V = 403.05
90	a = 8.4139 ± 0.0016, c = 5.6928 ± 0.0019	0.676	V = 403.01

**Table 2:** Comparative analysis of the shielding characteristics of CuBi_2_O_4_ films with calculated data obtained using the XCOM program code.

	Parameter			
	MAC, cm^2^/g	LAC, cm^−1^	∆_1/2_, cm^−1^	MFP, cm
**Theoretical (XCOM)**	1.55 ± 0.09	13.31 ± 0.23	0.052 ± 0.009	0.074 ± 0.005
**Experiment**	1.31 ± 0.13	10.94 ± 0.34	0.063 ± 0.007	0.091 ± 0.008

## Data Availability

Data are contained within the article.
